# A Systematic Review on Food Recommender Systems for Diabetic Patients

**DOI:** 10.3390/ijerph20054248

**Published:** 2023-02-27

**Authors:** Raciel Yera, Ahmad A. Alzahrani, Luis Martínez, Rosa M. Rodríguez

**Affiliations:** 1 Computer Science Department, University of Jaén, 23007 Jaén, Spain; 2 Computer Science Department, University of Ciego de Ávila, Ciego de Ávila 65100, Cuba; 3 Faculty of Computing and Information Technology, King Abdulaziz University, Jeddah 21589, Saudi Arabia

**Keywords:** food recommendation, diabetes, user preferences, nutritional information

## Abstract

Recommender systems are currently a relevant tool for facilitating access for online users, to information items in search spaces overloaded with possible options. With this goal in mind, they have been used in diverse domains such as e-commerce, e-learning, e-tourism, e-health, etc. Specifically, in the case of the e-health scenario, the computer science community has been focused on building recommender systems tools for supporting personalized nutrition by delivering user-tailored foods and menu recommendations, incorporating the health-aware dimension to a larger or lesser extent. However, it has been also identified the lack of a comprehensive analysis of the recent advances specifically focused on food recommendations for the domain of diabetic patients. This topic is particularly relevant, considering that in 2021 it was estimated that 537 million adults were living with diabetes, being unhealthy diets a major risk factor that leads to such an issue. This paper is centered on presenting a survey of food recommender systems for diabetic patients, supported by the PRISMA 2020 framework, and focused on characterizing the strengths and weaknesses of the research developed in this direction. The paper also introduces future directions that can be followed in the next future, for guaranteeing progress in this necessary research area.

## 1. Introduction

Recommender systems are artificial intelligence-based tools focused on providing online users with the information that best fits their preferences and needs in a search space overloaded with possible options [1]. Three main paradigms have driven the development of recommender systems: (1) content-based recommendation, (2) collaborative filtering-based recommendation, and (3) hybrid recommendation.

Content-based recommendations are focused on providing users with items that are similar to those consumed or preferred in the past by the same users, being centered on user profiling, item profiling, and profile matching for accomplishing this task [2]. In contrast, collaborative filtering is focused on the past preference of similar users, for generating a recommendation to the active one [3]. Therefore, it is centered on the application of neighborhood models for finding more similar users to the current one, or on the use of machine learning models for capturing the knowledge associated with the preferences of such neighborhoods, to employ it in the recommendation generation process [4]. Furthermore, hybrid recommender systems have been also proposed for combining content-based and collaborative filtering recommendations [1]. Taking as a base these central paradigms, other approaches for conceiving recommender systems have been developed, such as knowledge-based recommendation, context-aware recommendation, or social network-based recommendation [5].

Based on these approaches, recommender systems have been successfully used in several domains such as e-commerce, e-learning, e-tourism, or e-health [6], being a relevant component for the success of popular platforms such as Amazon, TripAdvisor, or Booking.com [5].

Regarding the e-health context, food recommendation is currently emerging as a relevant recommendation scenario, taking into account their close relationship with non-communicable diseases and personalized nutrition concepts [7]. Personalized nutrition has been defined as healthy eating advice, tailored to suit an individual based on genetic data, and alternatively on personal health status, lifestyle, nutrient intake and phenotypic data [8]. Because of the cost of genetic data management, research efforts on managing these alternative data have increased intensely in the last few years and several computational solutions have been proposed with this goal.

In the specific case of diabetes, several studies have shown that more favorable dietary patterns are associated with lower glycemic load in older adults [9]. Furthermore, low-carbohydrate, Mediterranean, and high-protein diets are suggested to be effective in improving various markers of cardiovascular risk in people with diabetes and should be considered in the overall strategy of diabetes management [10]. Therefore, a tailored personalized nutrition approach in this scenario could directly lead to patient safety, as well as a worldwide impact regarding that diabetes is a leading global health emergency estimated to cost US$827 billion per year, and in 2021 it was estimated that 537 million adults were living with diabetes (https://www.makingdiabeteseasier.com/es/diabetes-explicada/diabetes/panorama-mundial-diabetes-2021, accessed on 4 January 2023).

Even though in the last few years several works have been focused on the development of healthy food recommendations, *the rationale for the current review in the context of the existing knowledge* comes from the fact that in the specific case of the diabetes domain, it has been detected the lack of a comprehensive analysis of the recent advances specifically focused on suggesting appropriate meals, supported by the recommender systems paradigm. The *motivation* of the current survey is then connected to the necessity of performing a consolidated review on food recommendations for diabetics, to be used by researchers and systems developers, for benefiting patients and other final users. Furthermore, it has been detected research literature in the medical knowledge domain, related to nutritional guidelines for the diabetic patient, could serve as starting point for developing computational models for supporting this scenario. Therefore, the *goal* of the current survey is the identification of current strengths and weaknesses in nutritional RSs for diabetics, for contributing to establishing a path for developing new research with this goal.

Based on these issues, the current literature review *addresses the following research questions*:

- RQ1: What are the current state-of-art research works related to food recommendation systems specifically focused on diabetic patients?
  In this case, we are interested in exploring how the several developments in food recommendation using computational tools, have covered the specific area related to diabetic patients. Here it is worth mentioning that there have been several research works focused on food recommendations considering nutritional criteria. However, a preliminary analysis of such works suggests that they are usually developed from a general viewpoint, being able to introduce knowledge to manage several non-communicable diseases. However, it seems that there is a lack of work focused on specific diseases such as diabetes. Therefore, it is necessary to develop a deeper study for characterizing this issue.
- RQ2: Which are the computational methods and tools used in such developments?
  In this direction, it is necessary to characterize the computational methods and tools previously used in food recommendation systems focused on diabetic patients. This characterization would identify gaps with possible solutions to this specific problem, which have not been explored yet by the research community. Furthermore, it will provide a concise representation of the current research results, facilitating the definition of a path to improve them.
- RQ3: Which are the evaluation frameworks used for measuring the effectiveness of such approaches?
  The evaluation is a critical stage in RS research. Furthermore, in scenarios like food recommendation, offline evaluation is currently difficult due to the lack of appropriate datasets. Therefore, it is required the simulation of real users, or the development of online evaluations. It is then necessary to characterize the performed research regarding these criteria, in previous works specifically focused on food recommendations for diabetic patients.

**Figure 1:**
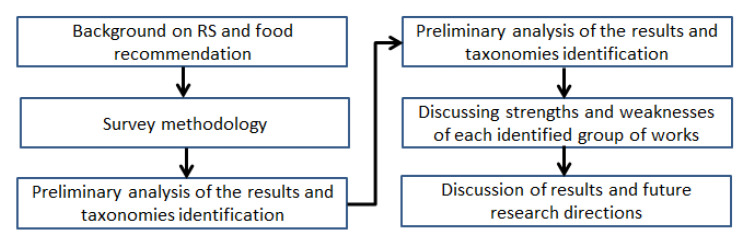
Flow of this paper.

The paper is structured as follows, depicted at Figure 1. Section 2 presents the necessary concepts linked to recommender systems and health-related food recommendations, usually used by the identified researchers. Section 3 presents the methodology used for performing this survey, based on the systematic literature review perspective. Section 4 presents the results, identifying the temporal distribution of the papers, and introducing a taxonomy for grouping them. Such taxonomy is composed of four groups, which are analyzed in detail, discussing the strengths and weaknesses in each case. An overall discussion of the obtained results is also included. Section 5 points out the next future research direction that could be developed in this area. Section 6 concludes the paper.

## 2. Background

This section is focused on presenting the basic concepts that are used in this survey. Specifically, it presents a background on recommender systems (Section 2.1), and health-aware recommender systems (Section 2.2).

### 2.1. Recommender Systems

A recommender system is considered as “any system that produces individualized recommendations as output or has the effect of guiding the user in a personalized way to interesting or useful objects in a large space of possible options” [1]. With this purpose, since the 1990s recommender systems have been developed taking as base two main tasks [11]: (1) the prediction task, focused on predicting the preference of a specific user over an unknown item, and (2) the recommendation task, focused on generating a list of top n preferred items also for a specific user.

Several taxonomies have been proposed for grouping recommender systems [1,5,12]. Such classification groups recommendation approaches into three main categories: (1) content-based recommender systems, (2) collaborative filtering-based recommender systems, and (3) hybrid recommender systems.

- *Content-based recommender systems.* These systems are focused on exploiting item features for building items and user profiles, which are used as a base for recommendation generation. From the general viewpoint, content-based recommendation comprises three phases [13]: (1) Item and user modeling, (2) User-item utility calculation, and (3) Recommendation generation. Based on this scheme, several content-based approaches have been developed, including simple approaches using the vector space model for representing users, as well as more complex models that incorporate semantic knowledge management for modeling the information associated with item features.
- *Collaborative filtering recommender systems.* On the other hand, collaborative filtering recommender systems are focused on the use of the overall user’s preferences for generating recommendations. They are based on the working principle of recommending to the active user those items that have already been preferred by other users similar to him/her. Collaborative filtering methods can be classified into two big groups: (1) memory-based collaborative filtering, which implements the collaborative filtering principle in a direct way through the use of similarity functions, and (2) model-based collaborative filtering, focused on the use of computational intelligence and optimization-based models for capturing the knowledge associated with the user preference values, and generating recommendations based on the former principle.
- *Hybrid recommender systems.* These systems combine the best features of collaborative and content-based filtering, managing item attributes for guaranteeing better item profiling, and also considering extensive processing of the user preferences. In practice, hybrid RS approaches are usually conceived as tailored solutions for particular recommendation domains. Burke [1] identified several paradigms for accomplishing such hybridization, including the weighted, the switching, and the cascading hybridization methods [14,15].

This work is focused on performing a survey on the development of recommender systems for supporting nutritional recommendations for diabetic patients. Most of the approaches presented in this survey, match with some of these three recommender systems categories.

### 2.2. Health-Related Food Recommender Systems

In the last few years, several authors have focused on proposing food recommendation approaches.

In order to summarize these works, Trattner and Elsweiler [16] develop an extensive survey on food recommender systems, identifying 25 research works focused on this goal. They group these works into several categories such as content-based methods, collaborative filtering-based methods, hybrid methods, context-aware approaches, group-based methods, and health-aware methods. Here the first five categories are centered on using the users’ rating values over foods, combined with different food information such as the associated ingredients. On the other hand, the health-aware methods generate food recommendations based on the users’ health problems and are conceived for improving their nutritional habits. Trattner and Elsweiler identify then several approaches that directly incorporate nutritional aspects into the recommendation approach, based on the difference between the calories that the user needs, and the calories in each food or recipe [17]. Other approaches employ post-filtering approaches to incorporate further nutritional aspects [18].

In parallel, Kumar et al. [19] enumerate 16 research works that propose food recommendation approaches, mainly focused on the direct use of basic recommendation approaches such as collaborative filtering or content-based methods. Abhari et al. [20] also developed a similar review on food recommender systems, but only focused in this case on the used artificial intelligence technique, such as genetic algorithms, multi-agent systems, self-organizing maps, or K-means clustering.

Trang-Tran et al. [7] have also developed a recent survey presenting an overview on recommender systems specifically focused on the healthy food domain. They consider four types of food recommender systems:

1. **Type 1:** Focused on recommending healthier recipes or food items which are most similar to recipes/foods that the user liked in the past, including works such as [21,22].
2. **Type 2:** Focused on recommending those items which have been identified beforehand by health care providers [23].
3. **Type 3:** Systems that generate recommendations on the basis of considering both criteria from Type 1 and Type 2 [24].
4. **Type 4:** Systems that generate group recommendations in which food items are consumed in groups and not by an individual [25].

Furthermore, Yera et al. [26] as part of their work, performed an analysis of recent papers on food recommendation, identifying two main research clusters. The first research cluster is composed of works focused on building complex information models as base for the personalized services. Here, they include works that support the generated recommendations by flow charts [27], ontologies [28,29], or a social semantic mobile framework [30]. The second cluster is focused on nutritional information processing instead of prioritizing the data modeling task. This cluster includes food recommendation approaches based on optimization techniques such as ant colony optimization [31] or the bacterial foraging approach [32], as well as other approaches using multi-modal data such as Ge et al. [17]. Furthermore, this analysis also identifies some works focused on exploiting the visual features of foods for modeling individuals’ nutritional expectations, dietary restrictions, and fined-grained food preferences [33].

Finally, Trattner et al. [34], as a chapter of the Recommender Systems Handbook 2022 [35], have identified four major food recommendation subdomains, which are: (1) health, (2) cooking, (3) grocery, and (4) restaurants. In the specific case of health-aware recommendation, they also referred to recent works focused on building hybrid models for food and recipe recommendations [36,37,38].

Beyond the number of works focused on food recommender systems and specifically on health-related food recommender systems, it is worthy to note the lack of works specifically focused on the diabetes domain [34]. In this way, it is necessary to perform further analysis in this domain, for identifying strengths, weaknesses, and potential directions for getting research progress. This is the goal of the current paper.

The next section will present the methodology used for performing this systematic literature review.

## 3. Methods

This section is focused on presenting the methodology executed for performing a systematic review of the literature on food recommender systems for diabetic patients. This survey is performed according to the PRISMA 2020 statement [39] (Preferred Reporting Items for Systematic Reviews and Meta-Analyses). With this goal in mind, we have followed the steps of the PRISMA 2020 checklist, for conducting the survey development. The main stages of this checklist are described in this section.

**Figure 2:**
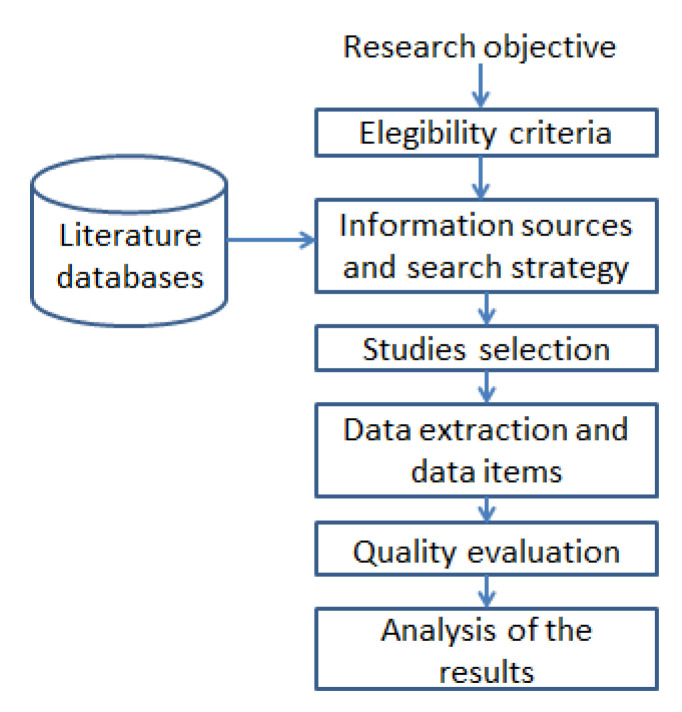
Search process for supporting the systematic literature review.

Figure 2 illustrates an overall perspective of the search process accomplished to reach the goal of this survey. It includes eligibility criteria, information sources and search strategy taking as input the literature databases, the studies selection, data extraction and data items, quality evaluation, and finally the analysis of the obtained results.

The current systematic literature review is driven by the following *inclusion and exclusion criteria*:

Inclusion criteria:

- Papers focused on food recommendations.
- Papers focused on presenting novel methods or software systems/architectures, that at least incorporate some feature related to personalization (i.e., providing tailored content according to each user’s characteristics)

Exclusion criteria:

- Papers focused on presenting medical information but without including any computational method or system.
- Papers that do not consider the diabetes dimension as a relevant feature of the proposal.
- Papers presenting only abstracts or small reports, with a lack of details on the proposed model.

The next subsections will describe in further detail the development of these stages.

### 3.1. Elegibility Criteria

### 3.2. Information Sources and Search Strategies

A literature search was performed on the first week on December 2022, using three electronic databases that have been used in previous reviews in related domains [40,41], which are Web of Science, Scopus, and PubMed (including Medline). In the case of Web of Science, it was specifically considered Web of Science Core Collection (http://webofscience.help.clarivate.com/Content/wos-core-collection/wos-core-collection.htm, accessed on 4 January 2023), including Science Citation Index Expanded, Social Sciences Citation Index, Arts & Humanities Citation Index, Emerging Sources Citation Index, and Conference Proceedings Citation Index. Studies from January 2010 to November 2022 were considered in the search process. Furthermore, the Cochrane Database of Systematic Reviews and the PROSPERO platform were also revisited to look for previous reviews focused on the current research questions, without finding any report centered on this theme.

The search was managed by taking as reference the main concepts linked to the research questions. In this way, it was considered:

- The direct use of recommender systems technologies for supporting food recommendations for diabetic patients: “food” AND (“recommender systems” OR “recommendation systems”) AND (“diabetics” OR “diabetes”).
- The use of further artificial intelligence-based personalization tools focused on nutrition for diabetic patient, that can be considered as recommender systems: “intelligent” AND “nutrition” AND (“diabetics” OR “diabetes”).
- Other kinds of interactive systems also focused on personalized food suggestions in diabetes: “food” AND “personalized” AND “systems” AND (“diabetics” OR “diabetes”).

### 3.3. Studies Selection

Primarily, the relevant articles were identified from the search output, based on the analysis of their title, abstract, and keywords. This process was performed by two reviewers (R.Y. and A.A.), taking as base the inclusion and exclusion criteria. Duplicated articles were removed, and the analysis of the full text of the remaining works was developed by two reviewers (R.Y. and L.M.). The remaining two reviewers (A.A. and R.M.) were also involved in the cases where further discussion was necessary to decide about the corresponding study inclusion or exclusion. In all cases, a final consensus was always reached.

### 3.4. Data Extraction and Data Items

In order to refine the inclusion criteria, several data were retrieved from the selected studies and tabulated in excel sheets. Three reviewers (R.Y., A.A., L.M) independently extracted the following data: authors, year, journal, main AI-based methodologies used, reported datasets, and level of the performed evaluation. Such information was synthesized, building a summary table that allows later the identification of four groups of research works based on the nature and the aim of the identified proposals (see below the results section). Here disagreements were also solved with discussions with the participation of the fourth reviewer (R.M.).

### 3.5. Quality Evaluation

Taking as base the approach recently used by Ataei and Litchfield [42], the quality evaluation of the identified manuscripts was performed through a specific checklist linked to software engineering and information systems research, instead of the direct use of well-established checklists for clinical studies, such as CASP [43] (Critical Appraisal Skills Programme) and I’s critical appraisal tool [44].

In this way, Ataei and Litchfield [42] developed a set of criteria made up of 7 elements, and informed by those proposed by CASP for assessing qualitative research [43], and also by the guidelines provided by Kitchenham et al. [45] in relation to empirical research in software engineering. Such categories and the corresponding criteria are:

(1) Minimum threshold quality:
  (a) Does the study report empirical research or is it merely a ‘lesson learnt’ report based on an expert opinion?
  (b) The objectives and aims of the study are clearly communicated, including the reasoning for why the study was undertaken.
  (c) Does the study provide adequate information regarding the context in which the research was carried out?
(2) Rigour:
  (a) Is the research design appropriate to address the objective of the research?
  (b) Is there any data collection method used and is it appropriate?
(3) Credibility:
  (a) Does the study report findings in a clear and unbiased manner?
(4) Relevance:
  (a) Does the study provides value for practice or research?

In our current review, these criteria were evaluated by the reviewers through dichotomous answers (i.e., yes or no), in two phases. The Minimum Threshold Quality area was assessed in the first phase. The second phase was then applied to the remaining areas, for those studies that passed the first phase. In the case of disagreements, small meetings with the reviewers were performed. Overall, the quality was agreed if at least 75% of the responses were positive for any retrieved work. Otherwise, the work was not considered for the current systematic review.

The next section will present an analysis of the results obtained through the application of the discussed literature review methodology.

## 4. Results

**Figure 3:**
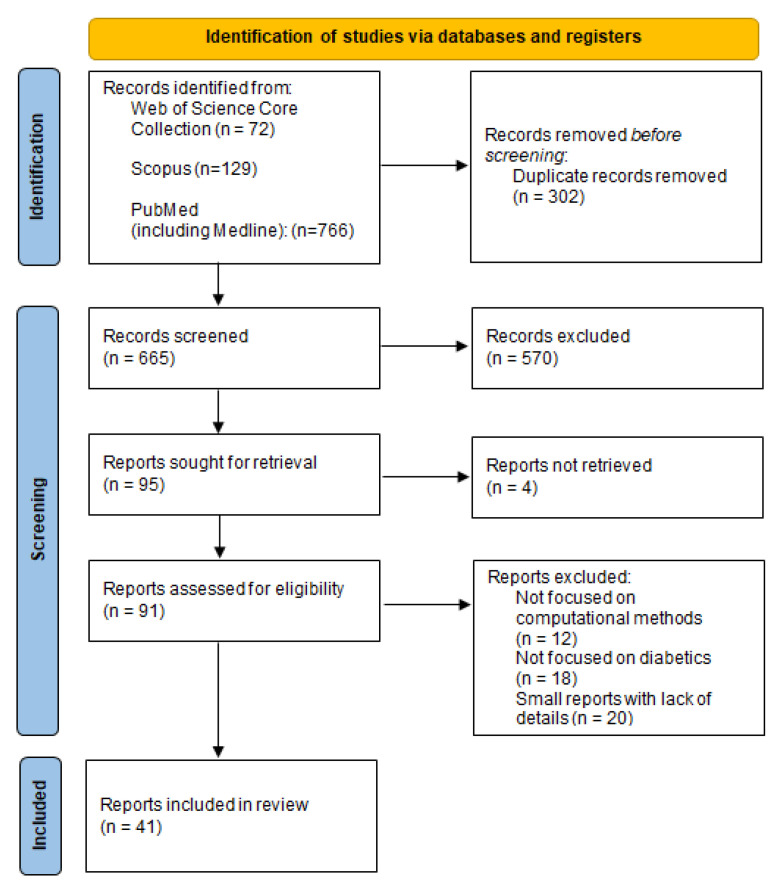
Systematic flow diagram representing the inclusion of studies according to the PRISMA 2020 Declaration.

A total of 967 records were initially detected using the methodology discussed in the previous section (Figure 3), distributed as follows in the different used databases:

- *Web of Science Core Collection:* The search procedure at this database obtains 72 research papers according to the search strings.
- *Scopus:* In this case the search procedure leads to 129 research papers. Here some of the research papers retrieved by Scopus were already obtained through the Web of Science Core Collection database.
- *Pubmed (including Medline):* Being that Pubmed is a database with a wider scope and inclusion strategies, the proposed search strings initially obtain 766 registries. Here it is important to mention that most of the entries reached through Web of Science and Scopus, were also obtained by Pubmed.
  Overall, 302 duplicated records were detected across the search process in the three databases, that were removed at this stage.

Once the duplicated files were removed, 665 records were selected for screening based on their title and abstract. For each considered literature database, this screening process has the following peculiarities:

- *Web of Science Core Collection:* The screening process identified some retrieved outcomes focused on physician guidelines/recommendations for diabetic patients, that clearly were not focused on exploring computational solutions. This kind of work was removed at this stage. It leads to 31 papers to be explored in the next stage.
- *Scopus:* In a similar way to the Web of Science, several papers not centered on providing food recommendation methods to diabetic patients, were removed. This screening process taking as basis title and abstract leads to 49 papers.
- *Pubmed:* In this case, most of the relevant papers formerly covered by this database, were already covered by Web of Science and Scopus. Furthermore, at this screening stage we also discard some papers focused on describing software usability studies for providing nutritional information to diabetes patients, but without the incorporation of any recommendation technology. At last, we retain 15 papers from this database.

Summarizing, this screening stage leads to a set of 95 papers to retrieve and assess for eligibility, 91 of which were finally able to be analyzed. This analysis concludes that 12 of such papers were not actually focused on computational methods, 18 were not actually focused on diabetics, and 20 were small reports with a lack of details mainly on the used methodology, dataset, and evaluation protocols. These papers were excluded from the review due to such reasons, finally leaving 41 articles to be considered at the end of this stage, as it is presented in Figure 3, based on the PRISMA 2020 statement.

The quality checklist discussed in the previous section is applied to such 41 articles. It discards works that were only focused on an initial screening of the related approach, but without a comprehensive description of their steps. In addition, they discarded some research works that lack a practical demonstration of their application; and therefore that does not reflect their clear value for practice and research. This analysis leaves a set of 34 papers that will be finally discussed in depth in this systematic review. The further subsection will analyze these papers, by grouping them through a proposed taxonomy.

### 4.1. Toward a Taxonomy for Grouping the Identified Papers

**Figure 4:**
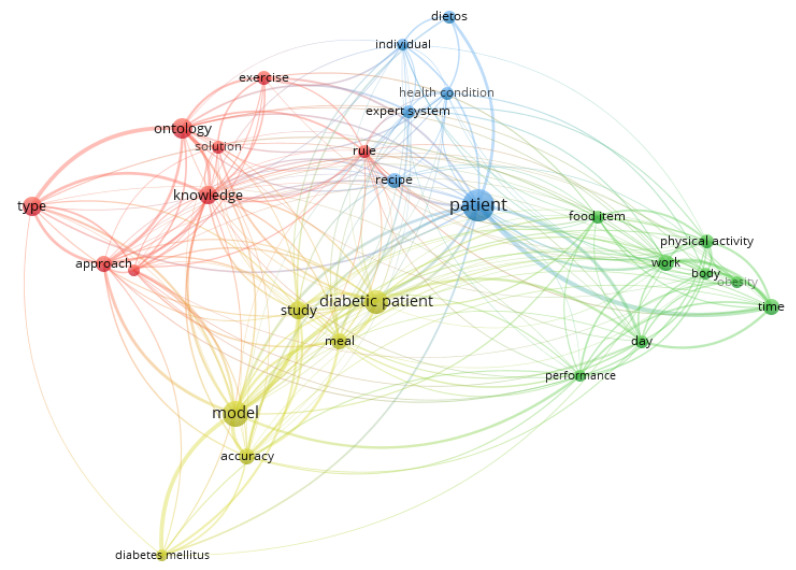
Relevant terms and their co-occurrence across the revised papers.

In order to introduce a taxonomy for grouping the identified papers, we explore the set of terms contained in their abstracts. Figure 4 globally illustrates the possible relationship between such terms, through their co-occurrence across the mentioned abstracts.

Particularly, we show the top 60% of the terms with a minimum number of occurrences of five, and those with larger values of co-occurrences are identified with the same colors. Each different color then represents a set of terms that appear together, and that therefore suggests the presence of a research trend around them.

As could be expected, Figure 4 highlights with a larger extent the terms that are associated with the main goal of this survey, such as “food”, “diabetes”, “health condition”, “patient”, etc. However, in the second stage, there are some additional terms that could suggest from a general viewpoint, some of the directions focused on facing the current research problem. At first, words such as “ontology” and “knowledge”, suggest the presence of works focused on semantic knowledge management. In the term co-occurrence graph, they are represented with the red color, and clearly represent a set of works that make use of semantic technologies for recommendation generation. The term “exercise” is also linked to this group of terms.

Furthermore, the location of terms such as “recipe”, “health condition”, “patient” and “expert system” in other groups, suggests the presence of AI-based works specifically focused on food recommendations for improving health and well-being in patients with chronic diseases, and that there are not directly linked to semantic knowledge management. This group of works is represented with the blue color in Figure 4.

The co-occurrence graph also identifies a third cluster (in green) that includes terms such as “physical activity”, “work”, “body”, "”obesity”, and “day”, suggesting the development of more user-centered research works supported by their interactive capabilities.

Finally, a fourth cluster is identified with the yellow color, including words such as “model”, “accuracy”, and “study”, coupled with the “diabetic patient” and “meal” words, which suggest the presence of a group of works focused on proposing computational models centered on accuracy (e.g., optimization-based models, classification-based models, or decision-based models) and intensively focused on the diabetes domain.

Based on these facts, we propose to use a taxonomy that divides the identified works into four main groups:

1. **Semantic-based approaches**, containing those approaches that integrate any kind of semantic knowledge management.
2. **Optimization-based approaches**, focused on integrating some approaches that use restriction-based or optimization-based models for food recommendation generation.
3. **Rules-based and classification-based approaches**, focused on presenting a diverse set of works that incorporates techniques such as classification, multicriteria decision making, fuzzy inference, or production rules; for supporting food recommendations. Such techniques come from the field of data mining, computational intelligence, and multicriteria decision analysis, and fulfill the current goal to a larger or lesser extent.
4. **Interaction-based approaches**, having as a common feature, the development of an interactive process with the users, for obtaining the final recommendations.

This taxonomy is related to previous taxonomies also proposed for grouping works in this area, such as the proposed by Yera et al. [26], which suggests the presence of two research branches in food recommendation applications:

- A group composed of works focused on building complex information models as the basis for personalized services, including semantic models. (In our taxonomy, category 1).
- A group of works centered on nutritional information processing that manages the available nutritional information sources instead of prioritizing the data modeling task. (In our taxonomy, the categories 2, 3 and 4).

The next subsections will analyze in detail, the works belonging to the four mentioned categories.

#### 4.1.1. Semantic-Based Approaches

Table 1 illustrates the identified works focused on the use of semantic technologies, 10 works in this group. The identified works have as common features the use of ontologies for managing the knowledge relevant to the nutrition domain. Furthermore, such ontologies are integrated with inference mechanisms such as decision trees or the Jena inference engine [46] for generating the recommendations. The use of case-based reasoning [47] was also identified with this aim.

As could be expected, most of the contributions in this group are developed through large software systems supporting the health and well-being of diabetic patients [48,49,50], with the food recommendations delivery as one of their dimensions.

**Table 1:** Summary of the identified related works using semantic technologies.

Papers	Key Feature	Evaluation Approach	Datasets
Lee et al. [51]	Propose a novel ontology model, which is based on interval type-2 fuzzy sets (T2FSs), called type-2 fuzzy ontology (T2FO), with applications to knowledge representation in the field of personal diet recommendation for diabetics.	Case studies	Not referred
Faiz et al. [50]	Implement an ontology-based integrated approach to combine knowledge from various domains, to generate diet and exercise suggestions for diabetics.	Not referred	Not referred.
Lo et al. [52]	The study is focused on an ontology-based dietary management system, which main contribution is to propose a method for synthesizing new recipes based on existing ones and to recommend appropriate recipes based on machine learning. It considers pathologies such as diabetes.	Focused on accuracy in the detection of inappropriate recipes.	Not referred
Chen et al. [46]	It is focused on diet recommendations for patients with chronic diseases including diabetes; using an ontology, decision trees, and the Jena inference engine.	Study with real participants, focused on accuracy	Data gathered in the user study
Yusof et al. [47]	It uses an ontological knowledge domain modeling based on a Malaysian food composition database. Furthermore, uses case-based reasoning for finding appropriate menus, identifying cases by the energy requirement, body mass index, religion, and race.	Not provided	Not provided
Alian et al. [48]	It integrates the users’ ontological profile with general clinical diabetes suggestions and guidelines, to make personalized recommendations (e.g., food intake and physical workout), based on the specific socioeconomic, cultural and geographical status. It is particularly focused on American Indian patients.	Two case studies, and criteria from medical experts	Not referred
Cioara et al. [49]	It defines dietary knowledge by nutritionists, encoding it as a Nutrition Care Process Ontology, and then uses it as the underlining base and standardized model for nutrition care planning. It provides personalized intervention plans covering nutrition education, diet prescription and food ordering adapted to the older adult’s specific nutritional needs, health conditions and food preferences	Use case validation	Not detailed
Selvan et al. [53]	It develops a fuzzy ontology-based recommender system using Type-2 fuzzy logic to recommend foods and drugs for chronic (diabetic) patients.	Case study	Not referred
Baek et al. [54]	It uses a hybrid clustering-based food recommendation method that employs chronic disease-based clustering, and a diet and nutrition ontology and knowledge base. User profiles are built according to their associated disease, including diabetes. Collaborative filtering is used to predict food preferences.	Offline experiments focused on RMSE performance	Food preference dataset gathered by the authors
Stefanidis et al. [55]	It presents a knowledge-based recommendation framework that exploits an explicit dataset of expert-validated meals to offer highly accurate diet plans across both healthy and unhealthy subjects with conditions including diabetes. It includes a qualitative ontology-based layer for verifying ingredient appropriateness and a quantitative expert-validated rules layer for synthesizing meal plans.	Comparison of the proposal’s generated daily meal plans and the experts’ recommendations.	Synthetic data with 3000 virtual profiles and their weekly meal plans

However, it is also worth mentioning that in these works it is very limited the development of evaluation approaches for measuring the outcomes of the proposals. Even though some works such as Chen et al. [46] develop studies with real participants focused on accuracy, most of them are limited to present demonstrative scenarios on the use of the proposal, usually supported by synthetic data [48,49,51]. In some cases, such as Alian et al. [48], Stefanidis et al. [55], medical experts are also used for validating such outcomes. Eventually, Lo et al. [52] is focused on presenting an approach for detecting inappropriate recipes for diabetics, and therefore centers its evaluation protocol on the accuracy of this task. Other works here, do not consider the use of any evaluation or demonstrative approach to the proposed methods.

This insufficient development of evaluation approaches leads to limited use of related datasets across the works, as well as a lack of data generation as the output of the proposals. Only Chen et al. [46], Baek et al. [54], and Stefanidis et al. [55] report the use of a dataset gathered in the user study they developed.

Summarizing, the analysis of this group of works leads to the identification of the following strengths and weaknesses:

**Strengths:**

1. The identification of a well-defined framework for research and development tasks around nutritional recommendations for diabetics using semantic technologies.

**Weaknesses:**

1. The lack of evaluation approaches for measuring the effectiveness of the proposals’ output.
2. Lack of generated datasets that limit the results’ reproducibility, affecting research progress.

#### 4.1.2. Optimization-Based Approaches

Our global analysis around food recommendations for diabetic patients also identified a group of works mainly focused on the use of optimization approaches for generating appropriate menus (Table 2). Here it is relevant to mention the use of integer programming models [26,56] or population-based metaheuristics [31,57,58] for formalizing constraints that guide to the reaching of nutritionally-appropriated menus. In the case of Pawar et al. [59], it is used a less formalized approach that considers a constraint satisfaction problem solved through forward checking algorithms for generating appropriate recipes.

In the analysis of these works, it is necessary to mention that even though the optimization approaches were pioneer methods in menu generation tasks [62], only in recent years can we identify a larger exploitation of their potential for menu generation in the diabetes scenario. Furthermore, in a similar way to the group that uses semantic technologies, here it can be identified the lack of formalized evaluation approaches and well-defined datasets. Here, five out of the eight analyzed works declare the use of simulation scenarios for measuring the performance of the proposal. In the case of Yera et al. [26], the authors report the use of a synthetic dataset. In other direction, the recent works of Jeyalakshmi and Poonkuzhali [61] and Salamah and Wardani [58] were focused on gathering datasets incorporating information from diabetic users, but at this moment with a lack of generalization.

Summarizing, here we can identify the following strengths and weaknesses:

**Strengths:**

1. The presence of established works with a well-defined use of specific optimization techniques for the menu generation for diabetics, could serve as starting point for further works.

**Weaknesses:**

1. Limited incorporation of knowledge related to diabetes disease in the proposed models. Here it is worth mentioning that even though there is a global effort of the research community around the menu recommendation problem using optimization techniques [7,32,63], there is a very limited effort specifically focused on the diabetes domain.
2. Lack of data that affects research progress.

#### 4.1.3. Rules-Based and Classification Approaches

A third group of works is focused on the use of classification or decision-making approaches for supporting nutritional recommendation delivery for diabetic patients (Table 3). Here we include several works with the use of IF-THEN rules as a component of an inference process that leads to the generated menus.

This group contains 10 works and presents a diversity of research that use rules which are in some cases extracted from real clinical datasets [64,65], and in other cases provided by the knowledge experts [66,67]. In the first case, it is relevant to use datasets with user information for supporting the proposals, specifically a clinical dataset on diabetes treatments [65], and data from the American Diabetes Association [64].

Other authors, such as Omisore et al. [68] use more sophisticated classification models like a neuro-fuzzy inference model for diabetes diagnosis, combined with a knowledge-based diets recommendation approach based on predefined menu templates, using information from the former neuro-fuzzy inference model. In this group, other researchers such as [67], also incorporate more basic fuzzy inference models.

Eventually, Sharawat and Dubey [69] uses the AHP approach for selecting an appropriate calorie food plan for diabetic patients; however, it just presents an initial screening, analysis and evaluation of the proposal, without additional details. In this way, in the previous group of works focused on optimization, it is worth mentioning that the work of Yera et al. [26] also incorporates an AHP-Sort approach for filtering out inappropriate foods for diabetic patients, as a previous step for the main optimization stage.

Furthermore, we also include in this group more consolidated research works focused on food recommendations, and that manage information centered on diabetic users to some extent. Here, Wang et al. [70] uses social network information from profiling users based on their health tags. Furthermore, they process such information for identifying appropriate recipes based on health tips. In other direction Nag et al. [71] is focused on restaurant dishes recommendation based on multi-modal data and users’ personalized health data stream, including diabetic users. In these works the authors use traditional offline metrics for evaluating recommendation generation; and also in both cases, they create datasets for evaluating the proposals.

Here we can identify the following strengths and weaknesses:

**Strengths:**

1. The development of consolidated research works such as [70,71], that present a full research cycle including evaluation and datasets generation. Even though they are not specifically focused on the diabetes domain, they can be extended and adapted with knowledge from such a domain.
2. The exploration in this scenario, of multi-criteria decision analysis, approaches such as AHP, which can be further used in the next future for supporting the nutritional recommendation of diabetics patients.
3. In contrast to the previously analyzed groups, here, 50% of works use some kind of dataset from measuring or demonstrating the use of the corresponding approaches.

**Weaknesses:**

1. In the case of research works using rule-based and inference systems, it cannot be appreciated a consolidated research direction, regarding it could be detected some overlapping in the contributions independently provided by the identified authors.
2. Overall, the static view of the output of systems contrasts with the dynamic nature of the underlying data.

**Table 3:** Summary of the identified related works using rule-based and classification approaches.

Papers	Key Feature	Evaluation Approach	Datasets
Lee et al. [72]	The system is designed to send information about the blood sugar levels, blood pressure, food consumption, exercise, etc., of diabetes patients. It manages the treatment by recommending and monitoring food consumption, physical activity, insulin dosage, etc.	Case study	Simulated data
Phanich et al. [73]	It proposes a food recommendation system by using food clustering analysis for diabetic patients. The system recommends the appropriate substituted foods in the context of nutrition and food characteristics. It uses experts for identifying normal, limited, and avoidable foods for diabetics, as well as an importance ranking of 18 nutrients for diabetics. It uses clustering for grouping appropriate foods characterized by such nutrients.	Scenario of use, and through questionnaires applied to nutritionists.	Not detailed.
Caballero et al. [66]	Propose a clinical decision support system focused on diet recommendation and insulin prescription, for gestational diabetes. The knowledge base was modeled with a traditional logic rule set consisting of IF-THEN production rules.	Initial study and clinical trial in terms of safety and effectiveness	Not declared
Nag et al. [71]	It presents a decision support system using multi-modal information based on timely, contextually-aware, and personalized data to find local restaurant dishes for satisfying a user’s needs. It takes nutritional facts regarding products, calculates which items are the healthiest, and then re-ranks and filters out the results based on the users’ personalized health data streams and environmental context. It considers different nutrient weights based on the current health condition, including diabetes.	Demonstrative working scenario	Food composition USDA dataset, and a created geo-tagged database with more than 10 million dishes from restaurants in California, USA.
Norouzi et al. [64]	Present a knowledge-based snack recommender system for diabetics, using constraint-based algorithms with inference rules obtained from the American Diabetes Association data.	Study with real participants, focused on evaluation questionnaires from patients and dietitians.	Data gathered in the user study
Sharawat et al. [69]	It uses the AHP method to find out the best diet for a diabetic patient, the quality judged on the basis of various qualifying factors such as body fat, burned calories, health carbs, and dietary needs	Working scenario	Not provided
Ramesh et al. [65]	Present a novel rule-based model for recommending foods for Indian elderly diabetic population based on Glycemic Index of food items. Rules are extracted using the Ripper algorithm from a real clinical dataset.	Use cases, evaluated by doctors.	Not declared.
Omisore et al. [68]	Present a multi-modal adaptive neuro-fuzzy inference model designed for the diagnosis of diabetes and a knowledge-based diets recommender model. The diets are based on predefined menu templates, tailored to the user preferences.	Case studies, and expert criteria	Private dataset with food prescriptions used for managing patients with diabetes.
Tabassum et al. [67]	It proposes a fuzzy inference system that receives input information such as gender, type of diabetes, age group, activity factor, and body mass index. It retrieves as output an appropriate calorie food plan, from a set of five predefined plans.	Not provided	Not provided
Wang et al. [70]	It uses a social network-based approach for building a health-aware user profile, also considering diabetic users. The recommendation then ranks the recipes based on the user’s health tags and the recipes’ nutritive value. With this goal in mind, for each user, it is constructed the positive and the negative samples from the recipe candidates according to the food-related health tips.	Offline protocol considering measures such as Hit Rate, NDCG, and AUC.	Dataset created by authors, made public.

#### 4.1.4. Interaction-Based Approaches

At last, we present a group of works that incorporate diverse approaches, but that have as a common feature, the development of an interactive process with users for obtaining the final recommendations (Table 4).

In this analysis, Table 4 suggests that the main goal of Ghosh et al. [74] is the development of a dedicated hardware system for quantifying physical labor during walking and running, using such information in a dynamic diet chart preparation system. In this way, they use an algorithm focused on incremental menu generation based on continuous interaction with the user and following the corresponding nutritional guidelines.

Similarly, the main goal of Sowah et al. [75] is the development of a diabetes question and answering chatbot that uses cognitive sciences for suggesting appropriate meals. This work is also supported by a neural network model and a k-nearest neighbors approach for meal recommendations.

**Table 4:** Summary of the identified related works using interaction-based approaches.

Papers	Key Feature	Evaluation Approach	Datasets
Agapito et al. [27]	Present a recommender system for the adaptive delivery of nutrition contents to improve the quality of life of both healthy subjects and patients with diet-related chronic diseases such as diabetes. It then generates nutritional recommendations based on user answers to dynamic real-time questionnaires.	Case studies	Private dataset
Sowah et al. [75]	It builds a Tensorflow neural network model for determining if a meal should be recommended for consumption, implements K-Nearest Neighbor (KNN) algorithm to recommend meals, and uses cognitive sciences to build a diabetes question and answer chatbot.	Demonstration of the developed system	Not provided
Ghosh et al. [74]	It describes a system that generates a dynamic diet chart based on calories spent by the body, and other data like the user’s body mass index and food preferences. It also presents a dedicated hardware system for supporting this information framework.	Case studies	Not referred
Mogaveera et al. [76]	Propose a system that aims at improving the health of patients suffering from various diseases by recommending them healthier diets and exercise plans by analyzing and monitoring health parameters. It uses a C4.5 decision tree for recommending and determining whether a particular food item and exercise should be given to a specific individual.	Evaluate the classification accuracy related to finding suitable food and exercises	Public datasets created by authors
Teixeira et al. [77]	It proposes a solution designed for diabetic people to find restaurants that are more suitable for their health needs. It uses a multi-agent architecture, incorporating case-based reasoning and sentiment analysis for benefiting the user’s lifestyle and health, including glycemic index analysis.	Evaluation of real scenarios	Not reported
Ribeiro et al. [78]	It presents SousChef, a meal recommender system that can help users to plan multiple meals considering the individual’s food preferences, restrictions, and nutritional needs including the case of diabetes. A greedy search approach is used for selecting the best recipe combination, by planning the meals one by one through the exploration of the search space with the most promising solutions.	Ad hoc protocol based on daily deviation from the ideal value of needed macronutrients, and recipe recommendation repetitions.	Synthetically generated data with simulated user profiles.

On the other hand, Agapito et al. [27] is focused on generating nutritional recommendations based on user answers to dynamic real-time questionnaires, which are used for building the profile of patients with diet-related chronic diseases such as diabetes. Based on such profiles, the authors suggest different foods from regional catalogs, based on their nutraceutical properties.

Finally, Mogaveera et al. [76] present a system focused on patients with diabetes, high blood pressure, or thyroid diseases. It is focused on two stages: health monitoring, and diet and exercises recommendations. In the second stage, they use a C4.5 decision tree classifier for determining the suitability of some diets and exercises for a specific patient.

Other works such as the developed by Teixeira et al. [77], specifically focused on diabetic patients, present ongoing research with a more limited scope. Finally, Ribeiro et al. [78] present a general framework that even though it includes an evaluation stage with synthetic users, does not sufficiently cover the scenario of diabetic patients.

The performed analysis leads to the following strengths and weaknesses in this group of works:

**Strengths:**

1. It is relevant that most of the works in this category gather information and develop the execution of their proposal in real scenarios, and two of them generated domain-related datasets.

**Weakness:**

1. The analysis identifies only two works specifically focused on the diabetes disease domain, and with a very limited evaluation and use of an appropriate database.
2. Less integration with computational intelligence and optimization tools, which could limit the performance of the current proposals with a larger volume of data.

The next section presents an overall discussion of the obtained findings, focused on analyzing the most established approaches, the research trends, and the main challenges.

### 4.2. Discussion

The analysis of the works discussed in this survey reflects a diversity of research approaches centered on food recommendation generation for diabetic patients.

**Figure 5:**
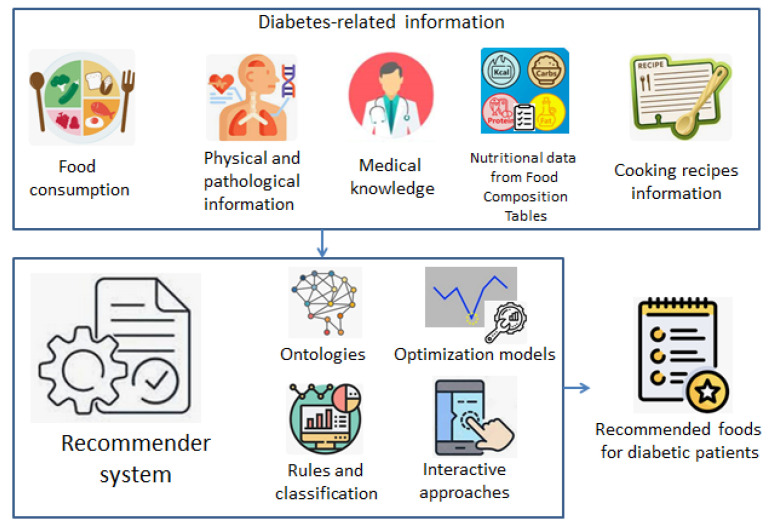
Overview of the data sources and recommendation methods identified at the survey.

Figure 5 illustrates an overview of the diabetic-related data that use recommender systems as input, according to the revised works. It includes previous food consumption logs and physical and pathological information such as insulin measurements, glucose levels, physical workouts, or blood pressure. Nutritional information coming from Food Composition Tables and recipe databases have been also considered across several identified works. At last, medical knowledge and guidelines have been also used to some extent in the food recommendation for diabetic patients.

The survey has also identified, according to Figure 5, that the main techniques used for building food recommender systems for diabetic patients were ontologies and semantic technologies, optimization methods, rule-based systems and classification methods, and approaches supported by the continuous interaction with the user. However, as will be explained below, these techniques have been also affected by several shortcomings associated with the generalization level, the limited incorporation of diabetes-related knowledge, or the lack of a common research and development framework.

At this moment, it could be considered that the default approach for facing this problem has been the use of semantic technologies with ontologies and rules for modeling the underlying knowledge, and performing reasoning to generate meal recommendations based on the diabetic patient’s conditions (Table 1). This review has identified works with different development scales, including research only focused on a screening and a preliminary evaluation of the proposed architecture [47,50], but also more consolidated works that incorporate a complete evaluation stage that presents then more evidence on their effectiveness to accomplish the initial goal [46]. Beyond this fact, it is important to take into account that knowledge representation through ontologies is a difficult task, and therefore even though this technology can be very effective in specific scenarios, it is not easy to reproduce the obtained results and generalize the developed solutions. This is a limitation for getting progress on the development of diabetes-aware food recommendation approaches considering the use of ontologies.

Our survey also identifies a set of works supported by the use of optimization methods for supporting menu generations (Table 2). Based on the former introduction of this kind of method for menu suggestions [62], in the last few years the traditional models focused on optimizing the menu’s calories considering the particular user needs, have been enriched with further disease-aware constraints including diabetes-related. Even though this group of works is more reproducible and generalizable in relation to semantic-based, a current limitation is related to the lack of datasets containing information about diabetic patients, that can be used for composing models specifically tailored to this disease. It is also remarkable that the activities focused on measuring the effectiveness of such approaches are centered on demonstrative cases and simulated scenarios, and in some scenarios, the model outcomes were checked by experts. However, it was not detected a complete evaluation protocol that can be systematically followed in future works for evaluating the output of the optimization-based food recommendation models.

This survey also identifies a group of works focused on the use of data mining approaches such as rules and classification, for recommendation generation in the current context (Table 3). Other kinds of works, such as those using fuzzy logic or multicriteria decision analysis, were also considered in this group. In this case, the analysis found several works with an important maturity level, such as Wang et al. [70] and Nag et al. [71], that incorporate novel food recommendation models, develop useful datasets, and present appropriate evaluation protocols. However, it is also important to mention that the most consolidated approaches do not consider the diabetes domain as the main component of the research, and only regard it as one of their dimensions. Other works, such as Tabassum et al. [67] and Omisore et al. [68] are more focused on the diabetics’ domain, but lack a detailed evaluation and use of an appropriate dataset. Here it is necessary to remark that it was also detected some works that incorporate diabetes-related knowledge but generate recommendations with a low personalized nature [72,73], due to the moderate use of the users’ preference values. Overall, this group of works presents very sparse research results that are far from a common research framework that would guarantee research progress in the next future.

This viewpoint is also applicable to the fourth identified group (Table 4). This group is composed of a smaller number of works and comprises research mainly focused on the development of interactive systems that use such interaction for gathering online information to be used in the recommendation generation [77,78]. However, the developed works are not mainly focused on building datasets with such information, to be used by the further research community working on the same research goal. Furthermore, it is not clear how these approaches can interact with the AI-based methods presented in the remaining three groups of work, for building more personalized solutions. In addition, in a similar way to the research works that incorporate semantic technologies, the identified research focused on interactive systems for food recommendation in diabetic patients were contextualized to specific scenarios, and therefore difficult to generalize.

The next section presents the advantages of the current literature review in relation to previous works with the same goal in mind.

### 4.3. Comparison with Other Reviews with a Similar Research Goal

In order to show the added value of the current literature review, Table 5 illustrates its contribution in relation to previous papers already mentioned in the Related work section and focused on food recommender systems.

Table 5 shows that previous literature reviews such as Kumar et al. [19] and Abhari et al. [20] are not centered on a well-formalized review methodology and analysis, difficulting reproducibility and future works projection.

In a different direction, more recent works such as Trattner et al. [16], Trang et al. [7], or Elsweiler et al. [34] are mainly focused on the use of diverse recommendation approaches in the food recommendation domain, without extensive analysis of the health-aware dimension and the diabetes-related knowledge.

The current work mitigates these research gaps, by presenting a detailed and reproducible literature review that is centrally focused on food recommendations for diabetic patients and is driven by the identification of a novel and useful taxonomy that can boost future research progress in this area.

The next section presents some future research directions that can be followed to fill the shortcomings identified across the paper.

## 5. Future Research Directions

The analysis of the strengths and weaknesses of the works focused on nutritional recommender systems for diabetic patients leads to the identification of several directions that should be followed in the next future, for guaranteeing progress in this necessary research area. The identified research directions can be:

1. **The definition of a consolidated framework to be used as a base for further research in nutritional recommendation generation for diabetics.** The developed analysis identifies the presence of parallel research lines with different strengths and weaknesses that can be complemented each other for building a more robust solution. In this way, it would be promising the integration of the optimization-based approaches (Section 4.1.2) and interaction-based approaches (Section 4.1.4), for exploiting the personalization capabilities of the interaction-based approaches, with the flexibility of the optimization-based approaches for modeling knowledge from this research domain.
2. **A better formalization of the research problem.** The analyzed works indistinctly focused the research efforts on individual food recommendations, menu recommendations, cooking recipe recommendations, or even restaurant dishes recommendations. In this way, it is necessary to unify as long as possible, the problem formalization and the expected output of these approaches, in the context of the nutritional recommendation for diabetic patients.
3. **A better exploitation of the knowledge domain for proposing computational solutions in this scenario.** Here it is necessary to exploit the more recent knowledge coming for the medical and the nutritional domain, related to the guidelines for healthcare and nutrition for the diabetic patient [79,80], for reflecting them into the developed computational models.
4. **A more intensive incorporation of user preferences in the recommendation generation.** Being recommender systems considered as applications focused on producing suggestions in a personalized way, it is important to reinforce the personalized dimension of the delivered recommendations in the current scenario. With this goal in mind, here it is important to consider the current user preferences and past foods consumption, also as input for the recommendation generation. However, most of the analyzed works use these information with a very limited extent, affecting the behavior of the develop approaches as a proper personalization/recommendation scenario.
5. **An extensive use of fuzzy tools in the nutritional recommendation approaches for diabetics.** Previous works have evidenced that the use of fuzzy tools in recommendation scenarios allows the development of more flexible and effective models in relation to the corresponding crisp alternatives [81,82]. Therefore, it is necessary to add the uncertainty management through fuzzy tools to the developed nutritional recommendation approaches for diabetics, as well as evaluating the effect of such tools in final recommender system output.
6. **The development of explainable recommendation approaches in this context**. According to the European Union Guidelines on Ethics in Artificial Intelligence (https://www.europarl.europa.eu/RegData/etudes/BRIE/2019/640163/EPRS_BRI(2019)640163_EN.pdf, accessed on 4 January 2023. AI systems and related human decisions are subject to the principle of explainability, according to which it should be possible for them to be understood and traced by humans. It is then necessary the development of intrinsic and post hoc recommendation explanation approaches [83,84], tailored to the particularities of the nutritional recommendation generation for diabetic patients.

## 6. Conclusions

The presented paper has been focused on developing a survey on the recent works centered on food recommender systems for diabetic patients. Even though in the last few years several authors have developed research works on food recommender systems, in the case of the diabetes domain they have not been conceived through a well-defined research line.

Based on such motivation, the current work develops a survey on food recommender systems for diabetic patients. After the initial identification of a set of papers with possible links to this topic from Web of Science, Scopus and Pubmed, 34 papers that have a direct contribution to this area were finally analyzed. Such papers were grouped through a taxonomy composed of four categories: (1) Semantic-based approaches, (2) Optimization-based approaches, (3) Rule-based and classification approaches, and (4) Interaction-based approaches. The developed analysis leads to the identification of strengths and weaknesses associated with each category, concerning the availability of datasets, the extent of the evaluation of the proposals, the integration level between approaches, the overlapping between the results proposed by several authors, and the lack of advances in research directions with potential to develop them; solving in this way the research questions presented at the beginning of the survey.

Future directions were pointed out, focused on the development of a consolidated framework for driving research in this area, a better exploitation of the knowledge domain for proposing computational solutions, a more intensive use of the user preferences for increasing personalization levels, and the addition of explanation capabilities to the developed models.

Furthermore, it is important to mention that this literature review remains pending some research issues that might be interesting to follow in the next literature analyses. As an example, the distinction between food recommendation approaches for type-1 and type-2 diabetes [85], and the potential for gathering useful information from IoT devices [86], to be used in food recommendation for diabetics.

With the current work, we hope to contribute to getting research progress in the development of food recommender systems for diabetic patient.

## Figures and Tables

**Table 2:** Summary of the identified related works using optimization approaches.

Papers	Key Feature	Evaluation Approach	Datasets
Pawar et al. [59]	Propose a recommendation system for recipes using a constraint knowledge-based recommendation method and a forward checking algorithm. It suggests recipes for diabetes disease.	Not detailed	Not detailed
Rehman et al. [31]	It presents a cloud-based food recommendation system, for dietary recommendations based on users’ pathological reports including diabetes. The model uses ant colony algorithms to generate an optimal food list and recommends suitable foods according to the values of the pathological report.	Simulation scenarios	Not provided
Sapri et al. [56]	Provide a combination of food menus that satisfy the daily nutrient requirements of a diabetic person with a minimum food spending, through an integer programming model.	Demonstrative example	Not specified
Yera et al. [26]	Present a general framework for daily meal plan recommendations for people with chronic diseases including diabetes, incorporating the simultaneous management of nutritional-aware and preference-aware information. Include a multi-criteria decision analysis-based pre-filtering step, and an integer programming step for generating the menus.	Ad-hoc criteria, such as previous frequency consumption, and overall preference over the suggested menu.	Synthetic data generated in the study.
Devi et al. [57]	It uses clustering for grouping patients according to features as age, weight, blood glucose level, etc. Furthermore, the improved Krill-Herd optimization is used to find the most appropriate neighborhood for generating nutritional recommendations.	Focused on prediction of insulin measurements and glucose levels	Patient profiles from UCI’s Diabetes dataset
Padmapritha et al. [60]	It presents a smart artificial pancreas for treating Type 1 Diabetes Mellitus in the elderly which simultaneously automates insulin administration but also the diet recommender system. The optimization-based diet recommender algorithm fuses the insulin infusion information of the model predictive control, long-term model, average carbohydrates and its variations to recommend diet and its patterns.	Case study using a real patient from a hospital	Data from a real patient.
Jeyalakshmi and Poonkuzhali [61]	It takes as input the user’s physical activity and the images of the preferred foods. Such information is brought into an optimization problem to determine the exact proportion of each food item, maximizing the Fullness Factor and minimizing the Glycemic Load of the overall diet.	Case study using gathered data	Data from diabetic users, gathered by the researchers.
Salamah and Wardani [58]	A food recommendation system was built to support a low protein diet for helping diabetic nephropathy patients by determining their daily food consumption. This research uses the Particle Swarm Optimization method to get the maximum variation of food ingredients.	Model output is evaluated by a clinical nutritionist	An Indonesian food database, and simulated user data supervised by a domain expert

**Table 5:** Summary of the identified related works using optimization approaches.

Previous Review	Main Features	Strengths of Our Current Review
Kumar et al. [19]	Enumerates 16 papers on food recommendation, mainly centered on the used RS technique. The search methodology is not explained. Not specific reference to diabetes.	Details a reproducible research methodology centrally focused on food recommendation for diabetes. Introduces a novel taxonomy that groups the identified research works according to used recommendation and machine learning approaches, indicating strengths and weaknesses at each case. Includes a comprehensive discussion section that is used as starting point for identifying future research directions.
Abhari et al. [20]	Mainly focused on exploring the used AI-based techniques. Lack of a comprehensive analysis of the literature for identifying strengths and weaknesses. Not specific reference to diabetes.	
Trang et al. [7]	Healthier food recommendations are covered with a less extent. Mainly focused on an overview of the identified works, without discussing the associated computational methods.	
Trattner et al. [16]	Mainly focused on the direct application of the RS approaches only supported by preference values in food recommendation. The incorporation of the nutritional information is slightly covered, and mainly focused on calories counting. Not focused on diabetes.	
Elsweiler et al. [34]	Identify four major food recommendation subdomains, which are: (1) health, (2) cooking, (3) grocery, and (4) restaurants. Not focused on the associated computational approaches and underlying taxonomies, that would support researchers for getting rapid progress. Not focused on diabetes.	
